# Selective Nitric Oxide Synthase Inhibitor 7-Nitroindazole Protects against Cocaine-Induced Oxidative Stress in Rat Brain

**DOI:** 10.1155/2015/157876

**Published:** 2015-10-20

**Authors:** Vessela Vitcheva, Rumyana Simeonova, Magdalena Kondeva-Burdina, Mitka Mitcheva

**Affiliations:** Laboratory of “Drug Metabolism and Drug Toxicity”, Department of “Pharmacology, Pharmacotherapy and Toxicology”, Faculty of Pharmacy, Medical University of Sofia, 2 “Dunav” Street, 1000 Sofia, Bulgaria

## Abstract

One of the mechanisms involved in the development of addiction, as well as in brain toxicity, is the oxidative stress. The aim of the current study was to investigate the effects of 7-nitroindazole (7-NI), a selective inhibitor of neuronal nitric oxide synthase (nNOS), on cocaine withdrawal and neurotoxicity in male Wistar rats. The animals were divided into four groups: control; group treated with cocaine (15 mg/kg^−1^, i.p., 7 days); group treated with 7-NI (25 mg/kg^−1^, i.p., 7 days); and a combination group (7-NI + cocaine). Cocaine repeated treatment resulted in development of physical dependence, judged by withdrawal symptoms (decreased locomotion, increased salivation and breathing rate), accompanied by an increased nNOS activity and oxidative stress. The latter was discerned by an increased formation of malondialdehyde (MDA), depletion of reduced glutathione (GSH) levels, and impairment of the enzymatic antioxidant defense system measured in whole brain. In synaptosomes, isolated from cocaine-treated rats, mitochondrial activity and GSH levels were also decreased. 7-NI administered along with cocaine not only attenuated the withdrawal, due to its nNOS inhibition, but also reversed both the GSH levels and antioxidant enzyme activities near control levels.

## 1. Introduction

Cocaine is a potent psychostimulant, recognized as one of the most significant examples of drug abuse due to intense feeling of euphoria and increased concentration and energy. The psychostimulant effects of cocaine appear to be mediated by its ability to enhance the dopaminergic activity in the mesocorticolimbic circuit through binding to the dopamine, serotonin, and noradrenalin transport proteins and directly prevent their reuptake into presynaptic neurons [1]. The repeat intake of cocaine is related to tolerance and development of dependence and serious injuries of the central nervous system, heart, and liver. One of the metabolic pathways of cocaine, N-oxidation mediated by cytochrome P 450 enzymes and flavin adenine (FAD) containing monooxygenases, leads to production of reactive oxygen species (ROS), namely, nitroxide, nitrosonium, and iminium ions, which have been recognized to be involved in cocaine-induced organ toxicity [2]. Increased reactive oxygen species production in the central nervous system has been identified to play a pivotal role in the neuropathology induced by drugs of abuse, including cocaine [3]. Oxidative stress may occur during or after drug exposure and/or during the withdrawal from the drug [4]. Not only has the role of nitric oxide and related N-methyl-D-aspartate (NO/NMDA) cascade been discussed in the process of development of tolerance and withdrawal from different drugs of abuse [5] but also it is considered an important source of oxidative stress, induced by psychostimulants [6]. Although nitric oxide (NO) plays an important physiological role as a neurotransmitter in the central nervous system (CNS), excessive neuronal nitric oxide synthase- (nNOS-) dependent NO release during high levels of NMDA receptor stimulation results in production of hydroxyl (HO^•^) and peroxynitrite (ONOO^−^) radicals that are responsible for oxidative injury [7].

7-Nitroindazole (7-NI) is a heterocyclic compound, which inhibits nNOS by competing with both L-arginine and tetrahydrobiopterin [8] and has been used extensively as a selective inhibitor of nNOS [9, 10]. There are a number of studies showing the beneficial effect of inhibiting nNOS activity as a means of reducing NO/NMDA-induced neurotoxicity and attenuating tolerance and withdrawal to psychoactive agents [9, 11, 12].

In the context of this information, the objective of the following study was to investigate the effect of 7-NI on withdrawal symptoms and neurotoxicity induced by multiple administration of cocaine to male Wistar rats.

## 2. Materials and Methods

### 2.1. Drugs and Chemicals

All the reagents used were of analytical grade. Cocaine, 7-nitroindazole, and other chemicals, sucrose, Tris, DL-dithiothreitol, phenylmethylsulfonyl fluoride, potassium phosphate, calcium chloratum (CaCl_2_), magnesium chloratum (MgCl_2_), L-arginine, L-valine, bovine hemoglobin, beta-nicotinamide adenine dinucleotide 2′-phosphate reduced tetrasodium salt (NADPH), ethylenediaminetetraacetic acid (EDTA), bovine serum albumin (fraction V), reduced glutathione (GSH), oxidized glutathione (GSSG), glutathione reductase (GR), cumene hydroperoxide, Percoll, and MTT (3-[4,5-dimethylthiazol-2-yl]-2,5-diphenyltetrazolium bromide), were purchased from Sigma Chemical Co. (Taufkirchen, Germany). 2,2′-Dinitro-5,5′-dithiodibenzoic acid (DTNB) and D-glucose were obtained from Merck (Darmstadt, Germany). Dimethyl sulfoxide (DMSO) was purchased from Valerus (Sofia, Bulgaria).

### 2.2. Animals

Male Wistar rats (6–8 weeks of age, body weight 200–250 g) were used. The rats were housed in Plexiglass cages (3 per cage) in a 12/12 light/dark cycle, under standard laboratory conditions (ambient temperature 20°C ± 2°C and humidity 72% ± 4%) with free access to water and standard pelleted rat food 53–3, produced according to ISO 9001:2008. Animals were purchased from the National Breeding Center, Sofia, Bulgaria. A minimum of 7-day acclimatization was allowed before the commencement of the study and their health was monitored regularly by a veterinary physician. All performed procedures were approved by the Institutional Animal Care Committee and the principles stated in the European Convention for the Protection of Vertebrate Animals Used for Experimental and Other Scientific Purposes (ETS 123) [13] were strictly followed throughout the experiment.

### 2.3. Experimental Design

Animals were divided into four groups (*n* = 12) as follows:

 group 1: control animals, treated with saline for 7 days, which were involved in the experiment from the very beginning and housed under the same standard laboratory conditions as the treated animals;
 group 2: animals, receiving 15 mg/kg^−1^ i.p. of cocaine for 7 days [14];
 group 3: animals, receiving 25 mg/kg^−1^ i.p. 7-NI for 7 days [10];
 group 4: animals, treated with 7-NI (25 mg/kg^−1^ i.p.) and 30 min later with cocaine (15 mg/kg^−1^ i.p.) for 7 days.

Twenty-four hours after the last administration of the compounds the animals were observed for behavioral changes related to the withdrawal syndrome. Then the animals were sacrificed through decapitation and brains were extracted. Brains of six animals from each group were taken for isolation of synaptosomes and brains from the other six animals of each group were used for measurement of nNOS and antioxidant enzymes.

### 2.4. Behavioral Observation Test

Quantitative assessment of behavioral changes in the animals was performed 24 hours after the last administration of cocaine, alone and in combination with 7-NI. The observations and changes were recorded on the basis of the standardized observation grid, derived from that of Irwin test [15], adjusted to the conditions and objectives of our study. Briefly, 24 hours after the last administration, six rats of each group were placed in separate cages and observed for 30 min. The rats were observed simultaneously with the control group, given vehicle for the following symptoms: decreased locomotor activity, excitation, changes in coordination, salivation, and respiration. The symptoms were evaluated by their presence or absence and were rated on a 3-point scale (slight, moderate, and marked). Being mainly the quantitative procedure, no formal statistical analysis was conducted.

### 2.5. Assessment of Biochemical Parameters in Whole Brain

The first part of our experiment was carried out in whole brain. Therefore no particular brain structures were identified and isolated. Briefly, the procedure we followed was as follows: after decapitation of six (*n* = 6) rats from each group, the brains were taken out, measured, and divided into four parts, one for measurement of nNOS activity, one for assessment of MDA quantity, one for GSH levels assessment, and one for measurement of antioxidant enzymes. The brain samples were consequently homogenized with the respective buffers.

#### 2.5.1. Preparation of Brain Tissue Extracts and Assessment of nNOS Activity

The brains were minced and homogenized in 10 volumes of buffer, containing 320 mmol L^−1^ sucrose, 50 mmol L^−1^ Tris, 1 mmol L^−1^ DL-dithiothreitol, and 100 *μ*g/L phenylmethylsulfonyl fluoride (pH = 7.2) according to the method used by Knowels and Moncada [16]. The homogenates were then centrifuged at 17 000 ×g for 60 min. The protein content was measured by the method of Lowry [17] with bovine serum albumin as a standard.

nNOS activity was measured spectrophotometrically using the oxidation of oxyhemoglobin to methemoglobin by NO. The incubation medium contained 40 mM potassium phosphate buffer, pH = 7.2, 200 *μ*mol L^−1^ CaCl_2_, 1 mmol L^−1^ MgCl_2_, 100 *μ*mol L^−1^ L-arginine, 50 mmol L^−1^ L-valine, 2.6 *μ*mol L^−1^ oxyhemoglobin, 100 *μ*mol L^−1^ NADPH, and brain extract. The change in the difference in absorbance at 401 nm and 421 nm was monitored with a double split beam spectrophotometer (Spectro UV-VIS Split), at 37°С. The activity of the enzyme was expressed in nmol/min/mg, using the millimolar extinction coefficient of methemoglobin 77.2 М^−1 ^cm^−1^.

#### 2.5.2. Preparation of Brain Homogenate for Assessment of Malondialdehyde (MDA)

The brains were homogenized with 0.1 М phosphate buffer and EDTA, рН = 7.4 (1 : 10). Lipid peroxidation was determined by measuring the rate of production of thiobarbituric acid reactive substances (TBARS) (expressed as malondialdehyde (MDA) equivalents) as described by Deby and Goutier [18] with slight modifications. Briefly one volume of the brain homogenate was mixed with 1 mL 25% trichloroacetic acid (TCA) and 1 mL 0.67% thiobarbituric acid (TBA). Samples were then mixed thoroughly, heated for 20 min in a boiling water bath, cooled, and centrifuged at 4000 rpm for 20 min. The absorbance of supernatant was measured at 535 nm against a blank that contained all the reagents except the tissue homogenate. MDA concentration was calculated using a molar extinction coefficient of 1.56 × 10^5^ M^−1^ cm^−1^ and expressed in nmol/g wet tissue.

#### 2.5.3. Preparation of Brain Homogenate for GSH Assessment

GSH was assessed by measuring nonprotein sulfhydryls after precipitation of proteins with trichloroacetic acid (TCA), using the method described by Bump et al. [19]. Briefly, brains were homogenized in 5% TCA (1 : 10) and centrifuged for 20 min at 4 000 ×g. The reaction mixture contained 0.05 mL supernatant, 3 mL 0.05 M phosphate buffer (pH = 8), and 0.02 mL DTNB reagent. The absorbance was determined at 412 nm and the results were expressed as nmol/g wet tissue.

#### 2.5.4. Preparation of Brain Homogenates for Antioxidant Enzyme Activity Measurement

Measured amounts of brain were rinsed in ice-cold physiological saline and minced with scissors. 10% homogenates were prepared in 0.05 M phosphate buffer (pH = 7.4) and centrifuged at 7,000 ×g and the supernatant was used for antioxidant enzymes assay. Analyses were performed in triplicate and the average values were taken. Protein content was measured by the Lowry method [17].

*(1) Catalase Activity (CAT)*. Catalase activity (CAT) was assessed following the method of Aebi et al. [20]. Briefly, 10 *μ*L of homogenate was added to 1990 *μ*L of H_2_O_2_ solution (containing 6.8 *μ*L of 30% H_2_O_2_ + 1983.2 *μ*L 0.05 M phosphate buffer, pH = 7.4). CAT activity was determined by monitoring the H_2_O_2_ decomposition which was measured spectrophotometrically by the decrease in absorbance at 240 nm. Enzyme activity was calculated using a molar extinction coefficient of 0.043/mM^−1^/cm^−1^ and expressed as *μ*M/minute/mg protein.

*(2) Superoxide Dismutase Activity (SOD)*. Superoxide dismutase activity (SOD) was measured according to the method of Misra and Fridovich [21], following spectrophotometric autoxidation of epinephrine at pH = 10.4, 30°C, using the molar extinction coefficient of 4.02/mM^−1^/cm^−1^. The incubation mixture contained 50 mM glycine buffer, pH = 10.4. The reaction is started by the addition of epinephrine. SOD activity is expressed as nmol of epinephrine prevented from autoxidation after addition of the sample.

*(3) Glutathione Peroxidase Activity (GPx)*. GPx was measured by NADPH oxidation, using a coupled reaction system consisting of glutathione, glutathione reductase, and cumene hydroperoxide [22]. Briefly, 100 *μ*L of enzyme sample was incubated for 5 minutes with 1.5 mL 0.05 M phosphate buffer (pH = 7.4), 100 *μ*L 1 mM EDTA, 50 *μ*L 1 mM GSH, 100 *μ*L 0.2 mM NADPH, and 1 unit of glutathione reductase. The reaction was initiated by adding 50 *μ*L cumene hydroperoxide (1 mg/mL) and the rate of disappearance of NADPH with time was determined by monitoring absorbance at 340 nm. Results were expressed as nmol/min/mg protein using the molar extinction coefficient of 6.22 mM^−1 ^cm^−1^.

*(4) Glutathione Reductase Activity (GR)*. GR was measured according to the method of Pinto et al. [23] by following NADPH oxidation spectrophotometrically at 340 nm and using an extinction coefficient of 6.22 mM^−1 ^cm^−1^. The incubation mixture contained 0.05 M phosphate buffer, pH = 7.4, 2.5 mM GSSG, and 125 *μ*M NADPH at 30°C. Results were expressed as nmol/min/mg protein.

*(5) Glutathione-S-transferase (GST)*. Glutathione-S-transferase (GST) activity was measured using 1-chloro-2,4-dinitrobenzene (CDNB) as the substrate [24]. The incubation mixture containing 1.6 mL 0.05 M phosphate buffer, 100 *μ*L 1 mM GSH, 100 *μ*L 1 mM EDTA, and 100 *μ*L homogenate was incubated for 15 minutes at 37°C. After the incubation, 100 *μ*L 1 mM CDNB was added and the increase in absorbance with time was recorded at 340 nm. Enzyme activity is measured using an extinction coefficient of 9.6 × 10^3^/M^−1^/cm^−1^ and is expressed as nmol of CDNB-GSH conjugate formed/minute/mg protein.

### 2.6. Isolation of Synaptosomes

Rats (*n* = 6 from each group) were decapitated, and the brains were taken out for synaptosomes isolation as described by Taupin et al. [25]. Briefly, the brains were homogenized in 10 volumes of cold Buffer 1, containing 5 mM HEPES and 0.32 M sucrose (pH = 7.4). The brain homogenate was centrifuged twice at 1000 ×g for 5 min at 4°C. The supernatant was collected and centrifuged three times at 10 000 ×g for 20 min at 4°C. The pellet was resuspended in ice-cold Buffer 1. The synaptosomes were isolated by using Percoll reagent to prepare the gradient and then were resuspended and incubated in Buffer 2, containing 290 mM NaCl, 0.95 mM MgCl_2_·6H_2_O, 10 mM KCl, 2.4 mM CaCl_2_·H_2_O, 2.1 mM NaH_2_PO_4_, 44 mM HEPES, and 13 mM D-glucose. Incubations were performed in a 5% CO_2_ + 95% O_2_ atmosphere. The content of synaptosomal protein was determined according to the method of Lowry et al. [17] using serum albumin as a standard. Synaptosomes viability was measured by mitochondrial activity (MTT reduction), described by Mungarro-Menchaca et al. [26]. The formed formazan crystals were dissolved in DMSO. The extinction was measured spectrophotometrically at *λ* = 580 nm. GSH levels in synaptosomes were assessed, using Ellman reagent (DTNB) [27], which forms color complexes with –SH group at pH = 8 with maximum absorbance at 412 nm.

## 3. Statistical Analysis

Statistical program “MEDCALC” was used for analysis of the data. The data are expressed as mean ± SEM of six rats in each group. The significance of the data was assessed using the nonparametric Mann-Whitney *U* test. Values of *P* ≤ 0.05 were considered statistically significant.

## 4. Results

### 4.1. Behavioral Observation Test

The effect of 7-NI on cocaine withdrawal symptoms is shown in Table 1. In the animals experiencing cocaine deprivation, the withdrawal was manifested by moderate decrease in locomotor activity, excessive salivation, discerned by dampness visible around mouth, and enhanced breathing. Deprivation of 7-NI did not induce any behavioral changes. In the animal group treated with 7-NI in combination with cocaine the withdrawal symptoms were attenuated. No changes in food and water consumption were observed.

Being an inhibitor of nNOS 7-NI administered alone led to a significant decrease in the enzyme activity by 40% (*P* < 0.05). Cocaine multiple administration resulted in statistically significant (*P* < 0.005) increase in nNOS activity by 59%. 7-Nitroindazole coadministration with cocaine restored the enzyme activity nearly to control levels. In addition, compared to cocaine only group, 7-NI decreased nNOS activity by 43% (*P* < 0.05). Results are shown in Table 2.

### 4.2. MDA Quantity and GSH Levels

A significant increase by 31% (*P* < 0.05) in MDA quantity and a marked decrease in GSH levels by 44% (*P* < 0.05) were observed in the brains of rats after 7-day administration of cocaine. Compared to the nontreated controls, 7-NI coadministration decreased MDA production and restored GSH levels. Compared to cocaine only group, 7-NI decreased MDA production by 21% (*P* < 0.05) and increased GSH levels by 64% (*P* < 0.05). Results are shown in Table 2.

### 4.3. Assessment of Antioxidant Enzyme Activity

**Figure 1:**
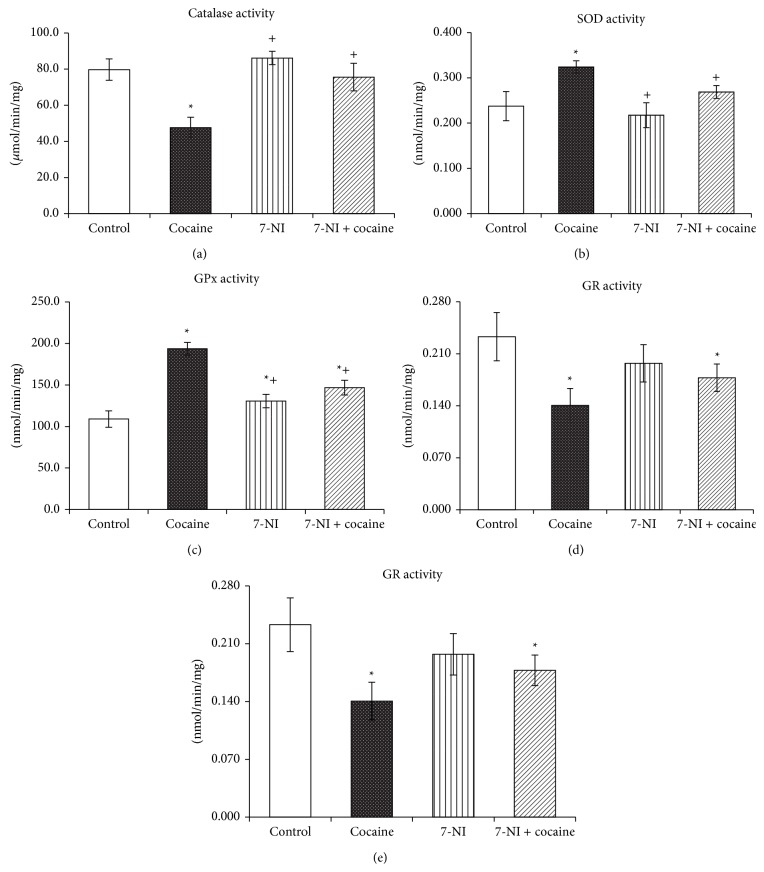
CAT, SOD, GR, GPx, and GST activity measured in rat brain from cocaine group (15 mg/kg^−1^, i.p., 7 days), 7-NI group (25 mg/kg^−1^, i.p., 7 days), and cocaine + 7-NI group. Parameters: CAT (catalase), SOD (superoxide dismutase), GR (glutathione reductase), GPx (glutathione peroxidase), GST (glutathione-S-transferase). Data are expressed as mean ± SEM of six animals (Mann-Whitney *U* test). ^*^Significant difference from control values (Mann-Whitney *U* test, *P* < 0.05); ^+^significant difference from cocaine-treated group (Mann-Whitney *U* test, *P* < 0.05).

The results are presented in Figure 1. Compared to the control group, cocaine toxicity is presented by increased activities of SOD (36%, *P* < 0.05) and GPx (78%, *P* < 0.05) and by decreased activities of other brain antioxidant enzymes, as follows: CAT (40%, *P* < 0.05), GR (39%, *P* < 0.05), and GST (45%, *P* < 0.05). Pretreatment with 7-NI prevented cocaine-induced toxicity by restoring the activities of antioxidant enzymes. When comparing the data obtained from 7-NI + cocaine group versus cocaine only group, a significant decrease in SOD activity by 17% (*P* < 0.05) and in GPx activity by 24% (*P* < 0.05) and a significant increase in CAT activity by 58% (*P* < 0.05) and in GST activity by 59% (*P* < 0.05) were observed.

### 4.4. Mitochondrial Activity (MTT Reduction) and GSH Levels

**Figure 2:**
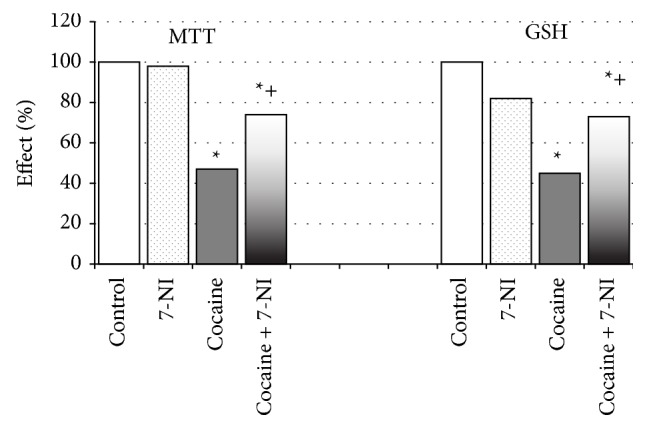
Effect of 7-nitroindazole (7-NI) in combination with cocaine on synaptosomal mitochondria activity (MTT reduction) and GSH level. Data are expressed as mean ± SEM of six animals (Mann-Whitney *U* test). ^*^Significant difference from control values (Mann-Whitney *U* test, *P* < 0.05); ^+^significant difference from cocaine-treated group (Mann-Whitney *U* test, *P* < 0.05).

The results of cocaine and 7-NI administration alone and in combination on mitochondrial activity, discerned by MTT reduction and GSH levels, showed that cocaine multiple treatment decreased MTT reduction by 53% (*P* < 0.05) and reduced GSH levels by 55% (*P* < 0.05). 7-NI alone administration did not change these parameters; however in combination with cocaine it showed protective effect discerned by preserved mitochondrial activity and GSH levels by 57% (*P* < 0.05) and by 60% (*P* < 0.05), respectively, compared to the cocaine only group. The results are depicted in Figure 2.

## 5. Discussion

Psychostimulants' abuse is a serious problem in the modern societies not only due to the development of drug addiction, which according to the criteria of the Diagnostic and Statistical Manual of Mental Disorders (DSM IV) [28] is considered a brain disease, but also due to the induced neurotoxicity. Cocaine is a well-known and widespread psychostimulant, notorious with its potential for tolerance and dependence development, as well as with its toxicity to the brain. One of the neurobiological mechanisms underlying these processes is thought to be activation of NMDA/NO cascade that results in an increase of nNOS activity and excessive production of NO that plays an important role both as a neurotransmitter and as a neurotoxicant [7]. In our study we investigated the possible neuroprotective effect of 7-NI, a selective inhibitor of the neuronal NOS after multiple cocaine administration alone and in combination with the inhibitor.

The results of our study indicate that repeated administration of cocaine for 7 consecutive days to male rats resulted in development of physical dependence, judged by withdrawal symptoms (decreased spontaneous locomotion, increased salivation and breathing rate), accompanied by an increased nNOS activity and development of oxidative stress. Cocaine exposure resulted in an increased formation of the lipid peroxidation product MDA and in the impairment of the nonenzymatic (GSH) and enzymatic (SOD, CAT, GR, GPx, and GST) potential in whole brain. In synaptosomes, isolated from cocaine-treated rats, the mitochondrial activity measured by MTT reduction and GSH levels were also decreased. Our results are in good correlation with the results of Bashkatova et al. [29], who observed high levels of lipid peroxidation in the hippocampus of rats exposed* in utero* to cocaine and with the observations of Poon et al. [30] that reported an oxidation of proteins in cocaine-exposed human neuronal progenitor cells. Moreover, the detected increased formation of thiobarbituric acid reactive substances (TBARS) (expressed as MDA) by cocaine corroborates the data obtained from the clinical study, carried out by Sordi et al. [31] in crack cocaine users during early withdrawal. The authors reported higher TBARS levels in severe crack users.

Cocaine exposure has been reported to increase hydrogen peroxide (H_2_O_2_) and lipid peroxide production in the prefrontal cortex and in the striatum of rats [32], brain structures with numerous dopaminergic nerve terminals. The present results showed that multiple cocaine administration led to impairment of the enzyme antioxidant defense, discerned by increased SOD and GPx activities and by a decrease in CAT, GR, and GST activities. In the available literature there are several proposed mechanisms underlying the changes of the oxidative status by cocaine. One of these mechanisms is related to the massive increase of dopamine release due to the cocaine binding to the transporter sites of monoamines that result in an inhibition of their uptake in the presynaptic neuron [1]. Enhanced neurotransmitter levels, primarily dopamine, in the synaptic cleft have been related to ROS formation [33]. Dopamine is further metabolized (by autooxidation) to generate hydrogen peroxide (H_2_O_2_) and superoxide anion (O_2_^−^) that can explain the observed increase in SOD and GPx activities. Our results support the data reported by Dietrich et al. [32] that determined an increase in SOD and GPx activities in brain cortex and striatum. At the same time cocaine administration decreased CAT, GR, and GST activities. Catalase is the main enzyme responsible for further detoxification of H_2_O_2_ to H_2_O and O_2_ [34]. The excessive amount of H_2_O_2_, produced on one hand by autooxidation of dopamine and on the other by cocaine itself, may explain the reduced CAT activity by the generated hydrogen peroxide. The increased GPx activity may be regarded as a compensatory mechanism in order to get rid of the excess peroxides due to lower CAT activity. The increased GPx activity is probably further responsible for the detected GSH depletion and decreased GR and GST activities.

The heterocyclic compound 7-NI that inhibits nNOS by competing with L-arginine and tetrahydrobiopterin [8] has been used extensively as a selective inhibitor of nNOS. Several studies have indicated that 7-NI affects different physical processes and behaviors, related to drug abuse, such as tolerance, withdrawal, neurotoxicity, psychomotor stimulation, and reward [35]. Our results showed that 7-NI administered along with cocaine attenuated the withdrawal, which is probably due to the detected decrease in nNOS activity, enhanced by cocaine (see Table 2). These data support the behavioral studies carried out by Haracz et al. [36] and Itzhak [9] in which the authors proved that administration of 7-NI reduced the hyperactivity and attenuated the induction of behavioral sensitization to cocaine. Along with its beneficial effects in drug addiction, antioxidant properties of 7-NI were also reported [37]. Chu et al. [38] found that 7-NI increased the SOD levels, decreased by hypoxic brain injury. In our study 7-NI administered along with cocaine decreased the formation of MDA and reversed both the GSH levels and antioxidant enzyme activities near control levels. Neuroprotective effects of 7-NI could be explained by its inhibition on nNOS activation and further formation of ONOO^−^, as well as by its MAO-B inhibition [39]. Furthermore, antioxidant properties of 7-NI, which are not nNOS dependent were suggested by Di Monte et al. [40] when MPTP (1-methyl-4-phenyl-1,2,3,6-tetrahydropyridine) treated mice showed a reduced brain MPP^+^ level after being injected with 7-NI.

## 6. Conclusions

Under the conditions of this study and on the basis of the obtained results we can conclude that in the experimental model of cocaine multiple administration in rats the selective nNOS inhibitor 7-nitroindazole not only attenuated the behavioral changes induced by cocaine deprivation but also exerted an antioxidant and neuroprotective activity. The possible mechanisms underlying the neuroprotective effects of 7-NI could be due to the combination of its inhibitory effect of nNOS and its direct free radical scavenging properties. The beneficial effect of 7-NI in restoration of the antioxidant cell defense in the brain, impaired by multiple cocaine administration, and along with it the attenuation of the physical dependence, induced by cocaine, once again confirm the role of the oxidative stress in the development of addiction to psychoactive compounds.

## Figures and Tables

**Table 1:** Quantitative assessment of some behavioral changes observed 24 hours after the last administration of cocaine and 7-NI.

Behavioral changes	Control	Cocaine	7-NI	7-NI + cocaine
Decreased locomotor activity	−	++	−	−
Excitation	−	++	−	−
Changes in coordination	−	−	−	−
Salivation	−	+++	−	+
Respiration-enhanced breathing	−	+++	−	+

*N* = 6 (for each treatment group).

+++ means severe; ++ means moderate; + means slight; − means no effect.

Observations were performed 24 hours after the last administration of the compounds. The symptoms were observed for 30 min. The observations and changes were recorded on the basis of the standardized observation grid, derived from that of Irwin test [15], adjusted to the conditions and objectives of our study.

**Table 2:** Changes in nNOS activity, MDA quantity, and GSH levels in brain homogenate after multiple administration of cocaine, alone and in combination with 7-NI.

Group	nNOS activity (nmol/min/mg)	MDA nmol/g/wet tissue	GSH nmol/g/wet tissue
Control	0.604 ± 0.04	3.55 ± 0.20	1.68 ± 0.15
Cocaine	0.935 ± 0.14^*^	4.65 ± 0.32^*^	0.94 ± 0.10^*^
7-NI	0.360 ± 0.07^*^	3.50 ± 0.10	1.59 ± 0.10
7-NI + cocaine	0.535 ± 0.10^+^	3.68 ± 0.15^+^	1.55 ± 0.08^+^

Data are expressed as mean ± SEM of six rats. ^*^Significant difference from control values (Mann-Whitney *U* test, *P* < 0.05); ^+^significant difference from cocaine-treated group (Mann-Whitney *U* test, *P* < 0.05).
